# Supplementary material for: Lewy body pathology exacerbates brain hypometabolism and cognitive decline in Alzheimer’s disease
Source: Nat Commun. 2024 Sep 14;15:8061. doi: 10.1038/s41467-024-52299-1 (PMC11401923; doi:10.1038/s41467-024-52299-1)
Supplement: Supplementary file 1 — Supplementary Information [file 41467_2024_52299_MOESM1_ESM.pdf]

Supplementary Information SAA ADNI

Supplementary Table-1. Baseline demographics by cognitive state

	Mild Cognitive Impairment (N=466)	Alzheimer's Dementia (N=329)	Whole cohort (N=795)
**Demographics**			
**Age, y**	74.4 (7.97)	75.8 (7.70)	74.9 (7.89)
**Sex (F, %)**	195 (41.8%)	129 (39.2%)	324 (40.8%)
**pTau181/Aβ_{42}-positive**	267 (57.3%)	305 (92.7%)	572 (71.9%)
**α-synuclein-positive**	98 (21.0%)	118 (35.9%)	216 (27.2%)
**AD/LB group**			
**AD-LB-**	168 (36.1%)	16 (4.9%)	184 (23.1%)
**AD-LB+**	31 (6.7%)	8 (2.4%)	39 (4.9%)
**AD+LB-**	200 (42.9%)	195 (59.3%)	395 (49.7%)
**AD+LB+**	67 (14.4%)	110 (33.4%)	177 (22.3%)
**Cognition**			
**MMSE**	27.7 (2.17)	21.9 (4.31)	25.3 (4.32)
**PACC**	-0.56 (0.47)	-1.68 (0.62)	-1.02 (0.77)
**Memory**	0.17 (0.60)	-0.91 (0.51)	-0.28 (0.77)
**Language**	0.38 (0.54)	-0.31 (0.65)	0.10 (0.69)
**Executive functioning**	0.35 (0.57)	-0.49 (0.74)	0.01 (0.77)
Missing	1 (0.20%)	1 (0.3%)	2 (0.3%)
**Visuospatial**	0.013 (0.38)	-0.34 (0.67)	-0.16 (0.57)
Missing	203 (43.6%)	60 (18.2%)	263 (33.1%)
**PET imaging**			
**FDG-PET**	1.21 (0.146)	1.03 (0.152)	1.14 (0.17)
Missing	51 (10.9%)	70 (21.3%)	121 (15.2%)

MCI: mild cognitive impairment; MMSE: Mini-Mental State Examination; PACC: Preclinical Alzheimer Cognitive Composite

* Represent presence of tremor or abnormal gait/motor strength; # Composite cognitive scores are in z-scores; $ Global AD ROI, as processed by the ADNI PET-core group.

Supplementary Table-2. Results Linear of mixed models using cognition as outcome

	AD-LB+*Time	AD+LB-*Time	AD+LB+*Time	AD-LB+*Time^2	AD+LB-*Time^2	AD+LB+*Time^2	ΔBIC
**Memory**<br>*N*=795, 3109 obs	-0.021 (0.04)<br>0.59	-0.19 (0.02)<br>< 2e-16	-0.21 (0.03)<br>4.91e-15	-0.01 (0.001)<br>0.025	-0.002 (0.003)<br>0.43	-0.009 (0.007)<br>0.19	-16.2<br>0.003
**Executive functioning**<br>*N*=793, 3070 obs	-0.01 (0.04)<br>0.84	-0.20 (0.02)<br>< 2e-16	-0.20 (0.03)<br>6.39e-10	-0.005 (0.01)<br>0.45	-0.005 (0.004)<br>0.19	-0.005 (0.01)<br>0.54	-16.3<br>7.632e-10
**Language**<br>*N*=795, 3100 obs	-0.06 (0.03)<br>0.032	-0.16 (0.02)<br>< 2e-16	-0.20 (0.02)<br>< 2e-16	na	na	na	+0.5<br>ns
**Visuospatial**<br>*N*=532, 1989 obs	-0.11 (0.04)<br>0.004	-0.11 (0.02)<br>1.12e-07	-0.12 (0.03)<br>3.60e-05	na	na	na	+24.9<br>ns

β *(SE), p-value* vs. reference group (AD-LB-); na: not available; ΔBIC: difference in BIC between two-sided linear and quadratic model. A negative value indicates preference for the quadratic model, while a positive term indicates preference for the linear model. P-value is from the model comparison chi-square statistic. Significant interactions between AD/LB group and time or time^2 reflect initial and accelerated decline in cognitive functioning, respectively. P-values are adjusted for multiple comparisons.

MMSE: Mini-Mental State Examination; PACC: Preclinical Alzheimer Cognitive Composite

Supplementary Table-3. Pairwise comparisons of linear of mixed models using cognition as outcome

	AD-LB+*time vs AD+LB-*time	AD+LB-*time vs AD+LB+*time	AD-LB+*time vs AD+LB+*time	AD-LB+*time^2 vs AD+LB-*time^2	AD+LB-*time^2 vs AD+LB+*time^2	AD-LB+*time^2 vs AD+LB+*time^2
**MMSE**	-1.22 (0.33) 0.0002	ns	-1.37 (0.36) 0.0002	ns	-0.15 (0.05) **0.005**	-0.19 (0.05) 0.002
**PACC**	-1.90 (0.47) 6.94e-05	ns	-2.05 (0.52) 0.0001	ns	-0.026 (0.008) **0.0015**	-0.029 (0.09) 0.001
**Memory**	-0.21 (0.04) 2.87e-08	ns	-0.23 (0.04) 1.96e-08	ns	ns	ns
**Executive functioning**	-0.19 (0.04) 7.58e-06	ns	-0.19 (0.05) 8.72e-05	ns	ns	ns
**Language**	-0.09 (0.03) 0.002	ns	-0.13 (0.03) 7.98e-05	na	na	na
**Visuospatial**	ns	ns	ns	na	na	na

β *(SE), p-value* vs. reference group (AD-LB-) derived from the two-sided linear mixed model; ns: not significant; na: not available; Significant interactions between AD/LB group and time or time^2 reflect initial and accelerated decline in cognitive functioning, respectively. P-values are adjusted for multiple comparisons.

Supplementary Table-4. Results Linear of mixed models using cognition as outcome for MCI population

	AD-LB+*Time	AD+LB-*Time	AD+LB+*Time	AD-LB+*Time^2	AD+LB-*Time^2	AD+LB+*Time^2	ΔBIC
**MMSE**<br>*N*=466, 2188 obs	0.024 (0.29)<br>0.93	-0.65 (0.15)<br>3.05e-05	-0.80 (0.24)<br>0.001	-0.012 (0.03)<br>0.69	-0.66 (0.15)<br>5.92e-06	-0.20 (0.05)<br>0.0002	-64<br><2.2e-16
**PACC**<br>*N*=466, 2188 obs	-0.02 (0.04)<br>0.62	-0.15 (0.02)<br>2.17e-10	-0.16 (0.04)<br>2.88e-05	-0.0003 (0.005)<br>0.95	-0.005 (0.002)<br>0.02	-0.03 (0.008)<br>0.0002	-49.2<br><2.2e-16
**Memory**<br>*N*=466, 2127 obs	-0.028 (0.03)<br>0.39	-0.16 (0.02)<br>< 2e-16	-0.19 (0.03)<br>1.83e-11	na	na	na	+15.5<br>0.005
**Executive functioning**<br>*N*=466, 2107 obs	-0.0004 (0.05)<br>0.99	-0.15 (0.02)<br>9.70e-10	-0.13 (0.01)<br>0.001	-0.006 (0.01)<br>0.46	-0.002 (0.004)<br>0.61	-0.016 (0.01)<br>0.18	-35.2<br>1.767e-13
**Language**<br>*N*=466, 2121 obs	-0.06 (0.04)<br>0.23	-0.12 (0.02)<br>5.46e-08	-0.14 (0.03)<br>7.44e-05	-0.002 (0.007)<br>0.83	-0.001 (0.003)<br>0.83	-0.011 (0.01)<br>0.19	-6.3<br>1.916e-07
**Visuospatial**<br>*N*=388, 1200 obs	-0.13 (0.03)<br>0.0002	-0.057 (0.02)<br>0.0013	-0.08 (0.03)<br>0.0039	na	na	na	+17<br>0.023

β *(SE), p-value* vs. reference group (AD-LB-); na: not available; ΔBIC: difference in BIC between two-side linear and quadratic model. A negative value indicates preference for the quadratic model, while a positive term indicates preference for the linear model. P-value is from the model comparison chi-square statistic. Significant interactions between AD/LB group and time or time^2 reflect initial and accelerated decline in cognitive functioning, respectively. P-values are adjusted for multiple comparisons.

MMSE: Mini-Mental State Examination; PACC: Preclinical Alzheimer Cognitive Composite

Supplementary Table-5. Pairwise comparisons of linear of mixed models using cognition as outcome for MCI population

	AD-LB+*time vs AD+LB-*time	AD+LB-*time vs AD+LB+*time	AD-LB+*time vs AD+LB+*time	AD-LB+*time^2 vs AD+LB-*time^2	AD+LB-*time^2 vs AD+LB+*time^2	AD-LB+*time^2 vs AD+LB+*time^2
**MMSE**	-0.67 (0.29)<br>0.02	ns	-0.82 (0.34)<br>0.016	-0.05 (0.03)<br>0.08	-0.13 (0.05)<br>0.012	-0.19 (0.06)<br>0.002
**PACC**	-0.13 (0.04)<br>0.03	ns	-0.14 (0.53)<br>0.010	ns	-0.03 (0.008)<br>0.002	-0.03 (0.01)<br>0.001
**Memory**	-0.13 (0.03)<br>7.42e-05	ns	-0.16 (0.04)<br>4.99e-05	na	na	na
**Executive functioning**	-0.15 (0.05)<br>0.0014	ns	-0.13 (0.05)<br>0.02	ns	ns	-0.02 (0.01)<br>0.08
**Language**	ns	ns	-0.09 (0.05)<br>0.08	ns	ns	ns
**Visuospatial**	0.07 (0.03)<br>0.03	ns	ns	na	na	na

β *(SE), p-value* vs. reference group (AD-LB-) derived from the two-sided linear mixed model; ns: not significant; na: not available; Significant interactions between AD/LB group and time or time^2 reflect initial and accelerated decline in cognitive functioning, respectively. P-values are adjusted for multiple comparisons.

Supplementary Table-6. Neuropathological scores across CSF-based AD/LB groups

	**AD-LB- (*N*=7)**	**AD-LB+ (*N*=4)**	**AD+LB- (*N*=27)**	**AD+LB+ (*N*=23)**
Age at death, y	82.6 (6.68)	88.3 (6.80)	81.0 (7.67)	80.5 (7.63)
Sex, F (%)	1 (14.3%)	2 (50.0%)	10 (37.0%)	3 (13.0%)
Measurement interval, y	3.14 (2.85)	5.25 (4.65)	5.22 (3.23)	3.26 (2.20)
Postmortem interval, hours	9.50 (7.35)	13.1 (6.68)	8.88 (8.71)	15.9 (16.4)
Lewy Body pathology				
No	7 (100%)	0 (0%)	20 (74.1%)	1 (4.3%)
Olfactory bulb	0 (0%)	1 (25.0%)	1 (3.7%)	0 (0%)
Brainstem/amygdala predominant	0 (0%)	1 (25.0%)	4 (14.8%)	6 (26.1%)
Cortical	0 (0%)	2 (50.0%)	2 (7.4%)	16 (69.6%)
Thal phases				
0	2 (28.6%)	0 (0%)	0 (0%)	0 (0%)
1	1 (14.3%)	3 (75.0%)	0 (0%)	0 (0%)
2	0 (0%)	0 (0%)	0 (0%)	0 (0%)
3	4 (57.1%)	1 (25.0%)	0 (0%)	1 (4.3%)
4	0 (0%)	0 (0%)	7 (25.9%)	9 (39.1%)
5	0 (0%)	0 (0%)	20 (74.1%)	13 (56.5%)
6	2 (28.6%)	0 (0%)	0 (0%)	0 (0%)
CERAD				
0	4 (57.1%)	3 (75.0%)	1 (3.7%)	2 (8.7%)
1	2 (28.6%)	1 (25.0%)	1 (3.7%)	2 (8.7%)
2	1 (14.3%)	0 (0%)	4 (14.8%)	2 (8.7%)
3	0 (0%)	0 (0%)	21 (77.8%)	17 (73.9%)
Braak stages				
0	1 (14.3%)	0 (0%)	0 (0%)	0 (0%)
1	2 (28.6%)	1 (25.0%)	0 (0%)	0 (0%)
2	2 (28.6%)	3 (75.0%)	1 (3.7%)	3 (13.0%)
3	2 (28.6%)	0 (0%)	1 (3.7%)	0 (0%)
4	0 (0%)	0 (0%)	0 (0%)	1 (4.3%)
5	0 (0%)	0 (0%)	16 (59.3%)	15 (65.2%)
6	0 (0%)	0 (0%)	9 (33.3%)	4 (17.4%)
TDP-43				
Hippocampus (present)	3 (42.9%)	1 (25.0%)	6 (22.2%)	5 (21.7%)
missing	0 (0%)	0 (0%)	0 (0%)	2 (8.7%)
Amygdala (present)	3 (42.9%)	1 (25.0%)	10 (37.0%)	8 (34.8%)
missing	0 (0%)	0 (0%)	0 (0%)	2 (8.7%)

Supplementary Table-7. Number of cases and observations for linear mixed model

	AD-LB-	AD-LB+	AD+LB-	AD+LB+
**MMSE**	*N*=164<br>898 obs	*N*=36<br>189 obs	*N*=350<br>1484 obs	*N*=160<br>590 obs
**PACC**	*N*=164<br>899 obs	*N*=36<br>190 obs	*N*=350<br>1489 obs	*N*=160<br>589 obs
**Memory**	*N*=164<br>856 obs	*N*=36<br>182 obs	*N*=350<br>1484 obs	*N*=161<br>590 obs
**Executive functioning**	*N*=164<br>852 obs	*N*=36<br>181 obs	*N*=349<br>1469 obs	*N*=159<br>576 obs
**Language**	*N*=164<br>853 obs	*N*=36<br>181 obs	*N*=350<br>1478 obs	*N*=161<br>588 obs
**Visuospatial**	*N*=103<br>416 obs	*N*=25<br>97 obs	*N*=266<br>1026 obs	*N*=137<br>450 obs

AD/LB group status and associations with domain-specific cognitive functioning

For the domain-specific cognitive composites, cross-sectional analyses showed that the AD+LB- (memory: β=-0.41, SE=0.05, p<0.001; EF: β=-0.27, SE=0.06, p<0.001; language: β=-0.16, SE=0.05, p=0.01) and AD+/LB+ (memory: β=-0.52, SE=0.06, p<0.001; EF: β=-0.50, SE=0.07, p<0.001; language: β=-0.35, SE=0.06, p<0.001) groups performed worse on most cognitive domains compared to the AD-LB- group (**Supplementary Figure-1**). Further, the AD+LB+ group exhibited worse scores on the executive functioning and language composites when compared to the other pathological groups, including the AD+LB- group (EF: β=-0.23, SE=0.06, p=0.0002; language: β=-0.19, SE=0.05, p=0.0013). For visuospatial functioning, lower performance of the AD+LB+ group was observed when compared to the AD+LB- group only (β=-0.18, SE=0.06, p=0.006).

Regarding cognition over time, LMMs with an additional quadratic term for time ($time^2$) were preferred for the memory and executive functioning domains based on ΔBIC, but not for language and visuospatial functioning. Both the AD+LB+ and AD+LB- exhibited faster decline across domains compared to the AD-LB- group but did not differ significantly from each other. In addition, the AD-LB+ exhibited faster decline in language and visuospatial scores compared to the AD-LB- group. On the contrary, while the AD-LB+ group showed no initial differences compared to the AD-LB- group in memory decline, they did exhibit an accelerated decrease over time ($\beta_{AD/LB\ group*time^2}$=-0.01, p=0.025, **Supplementary Figure-1, Supplementary Table-3/4**).

AD/LB group status and associations with cognitive functioning in the MCI population

LMMs including an additional quadratic term for time ($time^2$) were preferred for the MMSE and PACC based on ΔBIC. Over time, both the AD+LB- and AD+LB+ groups showed a significant initial (time) and accelerated (time2) global cognitive decline compared to the AD- groups (**Supplementary Table-5**). The accelerated decline was more pronounced for the AD+LB+ group than the AD+LB- group for both tests (**Supplementary Table-6**).

LMMs with an additional quadratic term for time (time^2) were preferred for executive functioning and language domains based on ΔBIC, but not for memory and visuospatial functioning. Both the AD+LB+ and AD+LB- exhibited faster decline across domains compared to the AD-LB- group, except for language functioning (**Supplementary Table-5**), but did not differ significantly from each other. (**Supplementary Table-6**). In addition, the isolated LB+ group demonstrated worse visuospatial performance compared to the reference population and pure AD group (**Supplementary Table-5/6 and Supplementary Figure-2F)**.

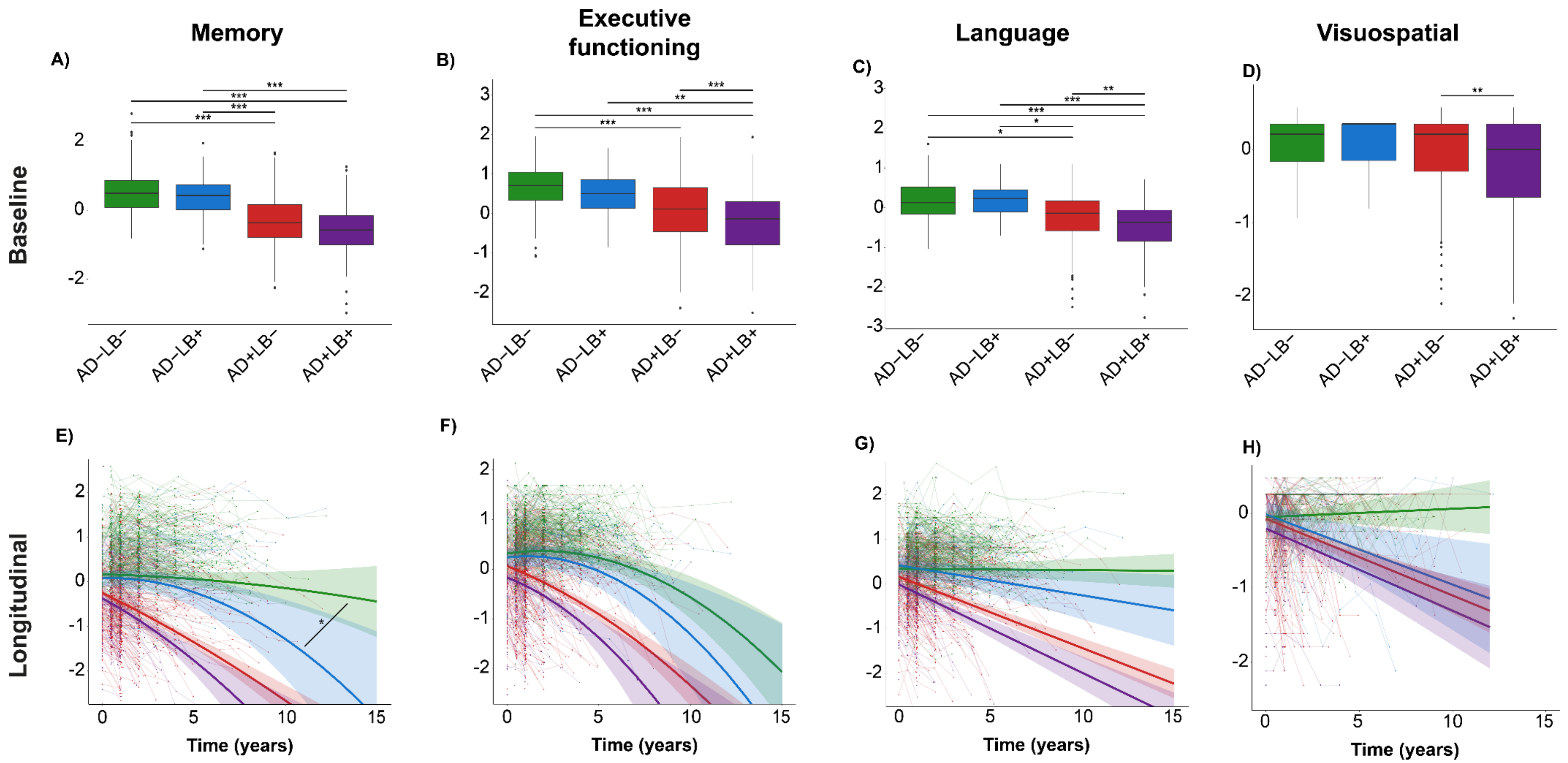

Supplementary Figure-1. AD/LB groups and cognitive domains

Figure illustrates AD/LB group differences for **A/C/E/J)** cross-sectional based on the two-sided GLM and **B/D/H/K)** longitudinal cognitive performance based on the two-sided LMM. All models were corrected for baseline age, sex, cognitive state, and level of education. Boxplots show the median, lower, and upper quartiles with whiskers representing minimum and maximum values. The spaghetti plots illustrate raw data regarding cognitive performance over time, while lines represent model fits (shaded area reflect 95% confidence interval). For memory and executive functioning domains, a model including an additional quadratic term for time better described the data, while for language and visuospatial functioning linear models were preferred. Lines within figure represent significant differences in AD/LB group*time^2. *$p_{adjusted}$<0.05, **$p_{adjusted}$<0.01, ***$p_{adjusted}$<0.001

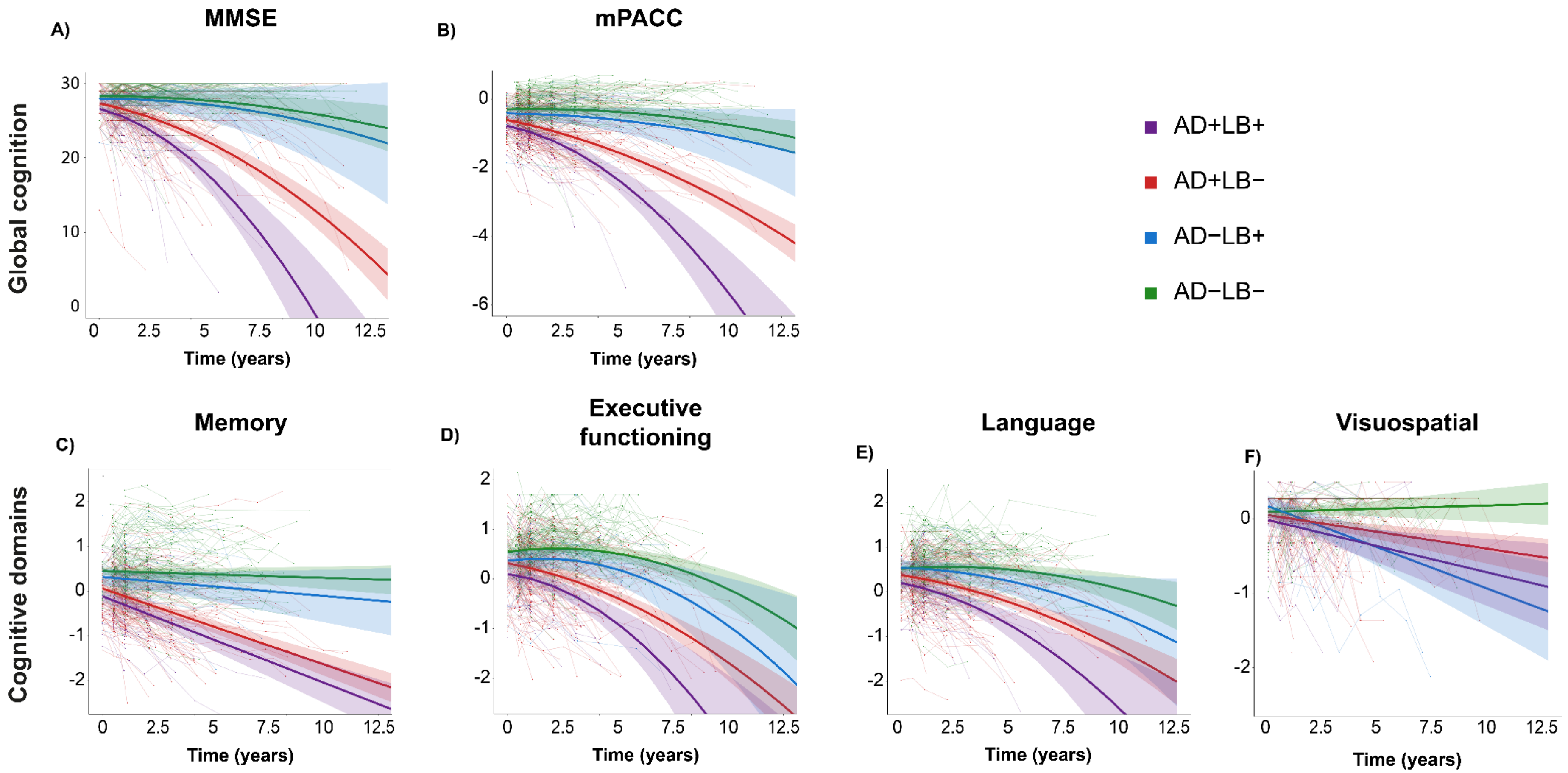

Supplementary Figure-2. AD/LB groups and cognitive domains over time in MCI population

Figure illustrates AD/LB group differences for longitudinal cognitive performance based on the two-sided LMM. All models were corrected for baseline age, sex, cognitive state, and level of education. The spaghetti plots illustrate raw data regarding cognitive performance over time, while lines represent model fits (shaded area reflect 95% confidence interval). For executive functioning and language domains, a model including an additional quadratic term for time better described the data, while for memory and visuospatial functioning linear models were preferred.

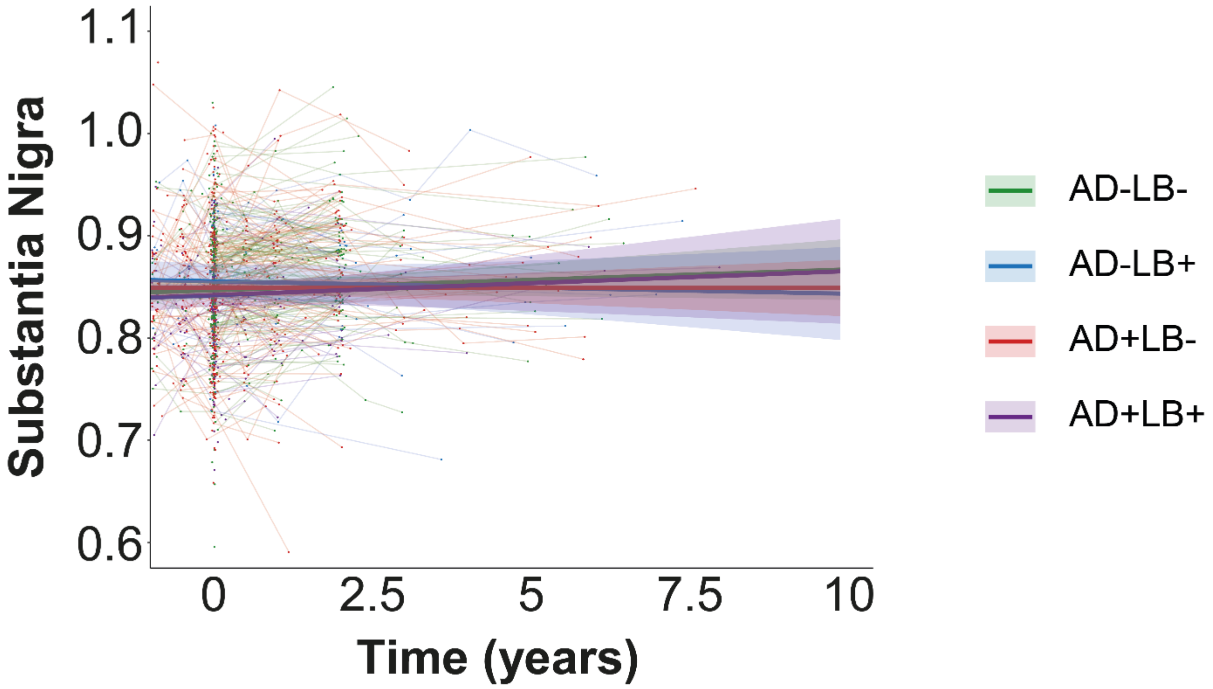

Supplementary Figure-3. **AD/LB group effect on Substantia Nigra metabolism over time.**

The spaghetti plots illustrate raw data regarding cognitive performance over time, while lines represent model fits (shaded area reflect 95% confidence interval).
