# Supplementary material for: Lewy body pathology exacerbates brain hypometabolism and cognitive decline in Alzheimer’s disease
Source: Nat Commun. 2024 Sep 14;15:8061. doi: 10.1038/s41467-024-52299-1 (PMC11401923; doi:10.1038/s41467-024-52299-1)
Supplement: Supplementary file 3 — Reporting Summary [file 41467_2024_52299_MOESM3_ESM.pdf]

nature portfolio

Corresponding author(s): Lyduine E. Collij

Last updated by author(s): 29-08-2024

Reporting Summary

Nature Portfolio wishes to improve the reproducibility of the work that we publish. This form provides structure for consistency and transparency in reporting. For further information on Nature Portfolio policies, see our Editorial Policies and the Editorial Policy Checklist.

Statistics

For all statistical analyses, confirm that the following items are present in the figure legend, table legend, main text, or Methods section.

n/a	Confirmed	
☐	☒	The exact sample size (*n*) for each experimental group/condition, given as a discrete number and unit of measurement
☐	☒	A statement on whether measurements were taken from distinct samples or whether the same sample was measured repeatedly
☐	☒	The statistical test(s) used AND whether they are one- or two-sided<br>*Only common tests should be described solely by name; describe more complex techniques in the Methods section.*
☐	☒	A description of all covariates tested
☐	☒	A description of any assumptions or corrections, such as tests of normality and adjustment for multiple comparisons
☐	☒	A full description of the statistical parameters including central tendency (e.g. means) or other basic estimates (e.g. regression coefficient) AND variation (e.g. standard deviation) or associated estimates of uncertainty (e.g. confidence intervals)
☐	☒	For null hypothesis testing, the test statistic (e.g. *F*, *t*, *r*) with confidence intervals, effect sizes, degrees of freedom and *P* value noted<br>*Give P values as exact values whenever suitable.*
☒	☐	For Bayesian analysis, information on the choice of priors and Markov chain Monte Carlo settings
☒	☐	For hierarchical and complex designs, identification of the appropriate level for tests and full reporting of outcomes
☒	☐	Estimates of effect sizes (e.g. Cohen's *d*, Pearson's *r*), indicating how they were calculated

Our web collection on statistics for biologists contains articles on many of the points above.

Software and code

Policy information about availability of computer code

Data collection	Data was retrieved from the ADNI database.
Data analysis	All analyses were performed in R Studio (version 4.2.2); package lme4, package emmeans, package MASS

For manuscripts utilizing custom algorithms or software that are central to the research but not yet described in published literature, software must be made available to editors and reviewers. We strongly encourage code deposition in a community repository (e.g. GitHub). See the Nature Portfolio guidelines for submitting code & software for further information.

Data

Policy information about availability of data

All manuscripts must include a data availability statement. This statement should provide the following information, where applicable:

- Accession codes, unique identifiers, or web links for publicly available datasets
- A description of any restrictions on data availability
- For clinical datasets or third party data, please ensure that the statement adheres to our policy

Data was retrieved from the ADNI database. The ADNI study was launched in 2003 as a public-private partnership led by principal investigator Michael W. Weiner, MD (adni.loni.usc.edu/).

Research involving human participants, their data, or biological material

Policy information about studies with human participants or human data. See also policy information about sex, gender (identity/presentation), and sexual orientation and race, ethnicity and racism.

Reporting on sex and gender	We used the term "sex" throughout the manuscript. Sex was determined based on self-reporting. Statistical analyses included sex as a covariate. In addition, we compared pathological groups regarding sex proportions. In line with the higher prevalence of Lewy body diseases in males, there was a strong tendency for more males in the AD-LB+ group (79.5% vs. 57.1%-60.5%, though this did not reach statistical significance.
Reporting on race, ethnicity, or other socially relevant groupings	This study did not include categorization of race, ethnicity and/or other socially relevant groupings.
Population characteristics	Detailed information is given in Table 1 for the clinical cohort and supplementary table 1 , and in supplementary table 6 for the post-mortem cohort. In short, we present results for analyses from the open-access ADNI cohort, including 795 cognitively impaired patients, clinically suspected due to AD, as well as 61 autopsy cases.
Recruitment	ADNI began in 2004 under the leadership of Dr. Michael W. Weiner, funded as a private-public partnership supported by the National Institute on Aging, the Foundation for the National Institutes of Health, the Alzheimer's Association, and dozens of companies. The ADNI study has operated continuously since then and is currently in its fifth phase, ADNI4.<br>New participants are recruited across North America during each phase of the study and agree to complete a variety of assessments over time. Results are then shared by ADNI through the USC Laboratory of Neuro Imaging (LONI)'s Image and Data Archive (IDA).<br>The ADNI study was designed to approximate an interventional clinical trial cohort. The standards and practices surrounding clinical trials are dynamic and have changed throughout the lifetime of the study, and as such the procedures surrounding enrollment within the ADNI cohort has continuously adapted to the latest trends in order to better serve its purpose as a model data set for trial design.<br>Participants are assigned to diagnostic cohorts upon enrollment in the ADNI study. This initial categorization is based on a thorough clinical diagnostic evaluation conducted during the screening visit.
Ethics oversight	All participants gave written informed consent (ClinicalTrials.gov registry numbers: ADNI GO: NCT01078636; ADNI 1: NCT00106899; ADNI 2: NCT01231971).

Note that full information on the approval of the study protocol must also be provided in the manuscript.

Field-specific reporting

Please select the one below that is the best fit for your research. If you are not sure, read the appropriate sections before making your selection.

[x] Life sciences [ ] Behavioural & social sciences [ ] Ecological, evolutionary & environmental sciences

For a reference copy of the document with all sections, see nature.com/documents/nr-reporting-summary-flat.pdf

Life sciences study design

All studies must disclose on these points even when the disclosure is negative.

Sample size	795 clinical cases and 61 neuropathological cases
Data exclusions	Included data within 1 year of primary measurement (CSF SAA). Cases with an intermediate SAA result were excluded (n=17). This information is provided in the methods section
Replication	not applicable as datasets with all required biomarkers (and particularly the CSF SAA marker) are not available beyond already published
Randomization	n/a
Blinding	n/a

Reporting for specific materials, systems and methods

We require information from authors about some types of materials, experimental systems and methods used in many studies. Here, indicate whether each material, system or method listed is relevant to your study. If you are not sure if a list item applies to your research, read the appropriate section before selecting a response.

Materials & experimental systems

n/a	Involved in the study
☒	☐ Antibodies
☒	☐ Eukaryotic cell lines
☒	☐ Palaeontology and archaeology
☒	☐ Animals and other organisms
☐	☒ Clinical data
☒	☐ Dual use research of concern
☒	☐ Plants

Methods

n/a	Involved in the study
☒	☐ ChIP-seq
☒	☐ Flow cytometry
☒	☐ MRI-based neuroimaging

Clinical data

Policy information about clinical studies
All manuscripts should comply with the ICMJEguidelines for publication of clinical research and a completedCONSORT checklist must be included with all submissions.

Clinical trial registration	All participants gave written informed consent (ClinicalTrials.gov registry numbers: ADNI GO: NCT01078636; ADNI 1: NCT00106899; ADNI 2: NCT01231971).
Study protocol	The ADNI study was launched in 2003 as a public-private partnership led by principal investigator Michael W. Weiner, MD (adni.loni.usc.edu/).
Data collection	The ADNI study was launched in 2003 as a public-private partnership led by principal investigator Michael W. Weiner, MD (adni.loni.usc.edu/).
Outcomes	Cognition, FDG-PET, and post-mortem observations

Plants

Seed stocks	*Report on the source of all seed stocks or other plant material used. If applicable, state the seed stock centre and catalogue number. If plant specimens were collected from the field, describe the collection location, date and sampling procedures.*
Novel plant genotypes	*Describe the methods by which all novel plant genotypes were produced. This includes those generated by transgenic approaches, gene editing, chemical/radiation-based mutagenesis and hybridization. For transgenic lines, describe the transformation method, the number of independent lines analyzed and the generation upon which experiments were performed. For gene-edited lines, describe the editor used, the endogenous sequence targeted for editing, the targeting guide RNA sequence (if applicable) and how the editor was applied.*
Authentication	*Describe any authentication procedures for each seed stock used or novel genotype generated. Describe any experiments used to assess the effect of a mutation and, where applicable, how potential secondary effects (e.g. second site T-DNA insertions, mosiacism, off-target gene editing) were examined.*
